# Behavioural Activation for Depression; An Update of Meta-Analysis of Effectiveness and Sub Group Analysis

**DOI:** 10.1371/journal.pone.0100100

**Published:** 2014-06-17

**Authors:** David Ekers, Lisa Webster, Annemieke Van Straten, Pim Cuijpers, David Richards, Simon Gilbody

**Affiliations:** 1 Durham University/Tees Esk and Wear Valleys NHS Foundation Trust, Department of Medicine, Pharmacy & Health, Durham University, Stockton on Tees, United Kingdom; 2 Department of Medicine, Pharmacy & Health, Durham University, Stockton on Tees, United Kingdom; 3 Department of Clinical Psychology, VU University Amsterdam, Amsterdam, The Netherlands; 4 School of Medicine, University of Exeter, Exeter, United Kingdom; 5 Hull York Medical School and Department of Health Sciences, University of York, York, United Kingdom; University of Groningen, Netherlands

## Abstract

**Background:**

Depression is a common, disabling condition for which psychological treatments are recommended. Behavioural activation has attracted increased interest in recent years. It has been over 5 years since our meta-analyses summarised the evidence supporting and this systematic review updates those findings and examines moderators of treatment effect.

**Method:**

Randomised trials of behavioural activation for depression versus controls or anti-depressant medication were identified using electronic database searches, previous reviews and reference lists. Data on symptom level and study level moderators were extracted and analysed using meta-analysis, sub-group analysis and meta-regression respectively.

**Results:**

Twenty six randomised controlled trials including 1524 subjects were included in this meta-analysis. A random effects meta-analysis of symptom level post treatment showed behavioural activation to be superior to controls (SMD −0.74 CI −0.91 to −0.56, k = 25, N = 1088) and medication (SMD −0.42 CI −0.83 to-0.00, k = 4, N = 283). Study quality was low in the majority of studies and follow- up time periods short. There was no indication of publication bias and subgroup analysis showed limited association between moderators and effect size.

**Conclusions:**

The results in this meta-analysis support and strengthen the evidence base indicating Behavioural Activation is an effective treatment for depression. Further high quality research with longer term follow-up is needed to strengthen the evidence base.

## Introduction

Depression is the most common mental disorder in community settings [1] and recent predictions state that by 2030 it will be the leading cause of disease burden in high-income countries [2]. NICE [1] promote the use of cognitive behavioural therapy (CBT) combining both behavioural and cognitive techniques. More recently a meta-analysis has suggested equivalence across most psychotherapies for depression [3]. If this is the case the idea of parsimony, using the least complex but acceptable theoretically derived treatment, may offer considerable benefit in terms of stability and distribution of the chosen intervention.

Behavioural Activation (BA) may be one such parsimonious treatment option. It uses the principles of operant conditioning through scheduling to encourage depressed people to reconnect with environmental positive reinforcement. Whereas more complex therapies such as CBT require 1–2 years of intensive training for therapists to acquire the wide range of competencies the relative small set of techniques necessary for effective delivery of BA may be possible to acquire after 5 days [4].

It has been 5 years since we conducted the searches for our previous two meta-analyses which indicated BA offered an effective and simple intervention in 16 and 17 randomised controlled trials respectively [5], [6]. This systematic review and meta-analysis updates our previous work exploring the effectiveness of BA as a psychological therapy for depression compared to usual care as we were aware that new studies had been conducted. In addition we explore the relationship of study level moderators such as therapist training level, delivery mode, multi-morbidity, number of sessions and severity with treatment effect. The review also adds to the current evidence base by extending the review to explore BA compared to anti-depressant medication.

## Methods

### Identification and Selection of Studies

We included studies identified in previous meta-analyses [5], [6] and cross referenced with on additional BA review [7]. In addition we searched a database of 352 psychotherapy studies of depression. This database has been used in a series of published meta-analysis examining depression (www.evidencebasedpsychotherapies.org) and has been described in detail elsewhere [8]. It is updated yearly using a systematic and comprehensive review of all published evidence (1966 to January 2013) and included 14,164 abstracts (3,638 from pubmed, 2,824 from psycinfo, 4,682 from embase, and 3,020 from the Cochrane Central Register of Controlled Trials). Reference lists of identified studies and meta-analyses were examined to ensure no studies had been missed. Finally key researchers in BA were contacted to identify any missed studies or studies in press.

### Inclusion Criteria

We included all randomised controlled trials for adult (≥16 years) patients with a primary diagnosis of depression who were treated in community or in-patient settings with BA. BA was defined as a behaviourally oriented time limited psychotherapeutic intervention including key elements of self-monitoring and activity scheduling. As BA is a relatively recent term used to describe this intervention we also included studies of behavioural therapy for depression if self-monitoring and activity scheduling were core elements of the intervention. Comparators consisted of a range of waiting list, placebo and usual care. We did not explore the comparative effectiveness of BA with other psychotherapies as this has been updated in other recent reviews [3], [9] [9], [10]. We also explored studies where BA had been compared with antidepressant medication. This comparison has been missing in previous reviews and represents an important consideration as antidepressants remain the most commonly received treatment for depression [11]. We included studies in any language to reduce the risk of potential publication bias.

Studies excluded were those which included participants with psychosis or bipolar disorder, substance misuse problems, cognitive impairment or without depression as a primary diagnosis.

### Study Level Moderators

Subgroup analyses were conducted to explore any potential dispersion across results. We investigated the moderating effects of:

- Group/Individual therapy
- Clinical/non clinical populations (i.e. student samples)
- Recruitment setting/approach
- Baseline depression severity
- Method of depression categorisation at assessment
- Level of therapist experience (psychotherapist/psychologist compared to specifically trained non specialist)
- Control type
- Number of sessions
- Quality of included studies.

In addition we explored the type of behavioural treatment employed in the study and if these were associated with effect. We examined the number of the elements currently considered core to BA (self-monitoring, activity scheduling, functional analysis, values assessment) included in each study as a continuous variable and if the treatment were considered simple BA (predominantly self-monitoring and scheduling) or complex BA (self-monitoring, scheduling plus additional behavioural components such as functional analysis and/or values focussed interventions). This subgroup analysis represented an important consideration as more complex BA studies have been excluded from recent reviews [9] as they were deemed to represent ‘third wave CBT’. This classification is not commonly accepted however and careful consideration of the cumulative effect of intervention components would represent useful new data relevant to this debate.

### Outcome Measures

Our primary outcome measure was depression symptom level, collected either via self-rated or via clinician-rated measures. Where studies included multiple symptom measures, all data were entered and the mean effect was calculated, so that each study provided one estimate of effect.

### Quality Assessment

Quality of studies was rated according to the Cochrane Collaboration’s Tool for Assessing Risk of Bias [12]. The elements used were;

1. Adequate generation of randomisation sequence
2. Allocation concealment
3. Blinding of assessment
4. Dealing with missing data.

Due to the difficulties of blinding participants, therapists and other associated health professionals in psychotherapy studies, this quality factor was excluded. Each study was scored against the above to provide a score of between 0 and 4.

### Data Extraction and Sub Group Coding

Two researchers extracted data from each trial post treatment and where possible at follow up. Those data were checked by LW and DE in a series of meetings. Any inconsistencies were referred back to the original text. Missing data were requested from study authors by email. Missing standard deviation (SD) scores were imputed from other relevant studies where these data were not available, with imputations tested in sensitivity analysis as per accepted procedures [13]. Finally extracted data were reviewed in a group meeting (DE, LW, A VS and PC) where consensus was reached.

### Meta-analyses

Effect size was calculated using the Comprehensive Meta-analysis (version 2.2.064) [14] computer program using standardised mean difference (SMD) with value ranges of small (0–0.32), medium (0.33–0.55) and large (0.56 and above) as per standard convention [15]. This approach allows analysis of the same outcome (depression symptom level) using different scales by subtracting the post-test mean of the intervention group from the post-test mean of the control group and dividing results by the pooled standard deviation. This provided the SMD, a consistent scale across measures of depression symptom level in included studies. Hedges *g* was reported to adjust for potential small sample bias anticipated in this review. Where studies included two or more measures of depression, all data were entered and the mean effect size was calculated within the CMA program. Where studies reported stratified results (i.e. high/low severity) these were combined using the study as the unit of analysis in CMA to reduce undue influence on heterogeneity. A hierarchy of reported data was used for entry into meta-analysis, with means and standard deviations taking priority, as these were considered the best assessment of outcome. Where these were not reported we used effect size data, dichotomous data or tests of significance in that order of preference. Where studies reported dichotomous outcomes, data were used to calculate a standardised effect size using a logit transformation in CMA. We present pooled data with 95% confidence intervals. As we were including studies across a long time span and number of control conditions we anticipated heterogeneity, and hence calculated effect sizes using a random effects model [16]. The random effects model takes into account both within- and between-study variance. Statistical heterogeneity was examined using the I^2^statistic for statistical variation across studies [17]. The I^2^statistic provides a measure of the proportion of dispersion of effects across studies that reflect real differences rather than random error. Benchmark values of 25%, 50% and 75% reflect low, moderate and high heterogeneity respectively and we report with 95% confidence intervals. The I^2^statistic does not include a test of significance so we calculated the Q statistic and report P values associated with that. In addition, SMDs were translated into number needed to treat (NNT) using accepted formulae [18] to ease interpretation of results from a clinical perspective. NNT indicates the number of patients requiring intervention to achieve one additional positive outcome over a comparator.

Subgroup analyses were conducting using a mixed effects model [14], [19]. This process pools results within groups using a random effects model, and tests for significant difference between subgroups using a fixed effects model. Meta -regression was used for exploration of the moderating impact of continuous variables on effect size indicated by a Z-value and associated p value [14], [19]. We examined the impact of our *a priori* moderators and type of control condition on effect size. Publication bias was assessed through visual inspection of a funnel plot graph on the primary outcome (post-treatment depression score) for asymmetry. This is an accepted approach, but is subject to inconsistency, with sufficient studies (≥10) being required to differentiate real from spurious asymmetry [20]. In order to counter this problem, an Egger weighted regression test [21] was calculated to quantify potential publication bias, and the trim and fill procedure [14], [19] used to estimate effect size after any such bias was taken into account.

## Results

After examination 44 of the identified studies were excluded. The reasons for exclusion of these 44 studies were as follows: three studies did not randomise participants adequately [22]–[24], eleven only included active intervention comparisons (therefore no control/active control) [25]–[35], five studies reported excessively high attrition rates (≥50%) or incomplete outcome data [36]–[40] In two studies depression was not reported as the primary diagnosis [41], [42], three studies were excluded as participants suffered from primary substance misuse problems (drug/alcohol)[43]–[45]; one study was excluded as participants had a cognitive impairment [46]. Eight studies were excluded due to cognitive or counselling elements being included in the BA [47]–[54] and five studies were excluded as the symptom level measure used were not depression specific (e.g. BADS/HADS) [55]–[58]. Three studies were dissertation abstracts or papers that were not available for download in the UK [59]–[61]; one study was excluded as it was a pilot evaluation of culturally adapted behavioural activation [62] and finally two studies was excluded as they were doctoral dissertation versions of a later included published papers [63], [64].

**Figure 1:**
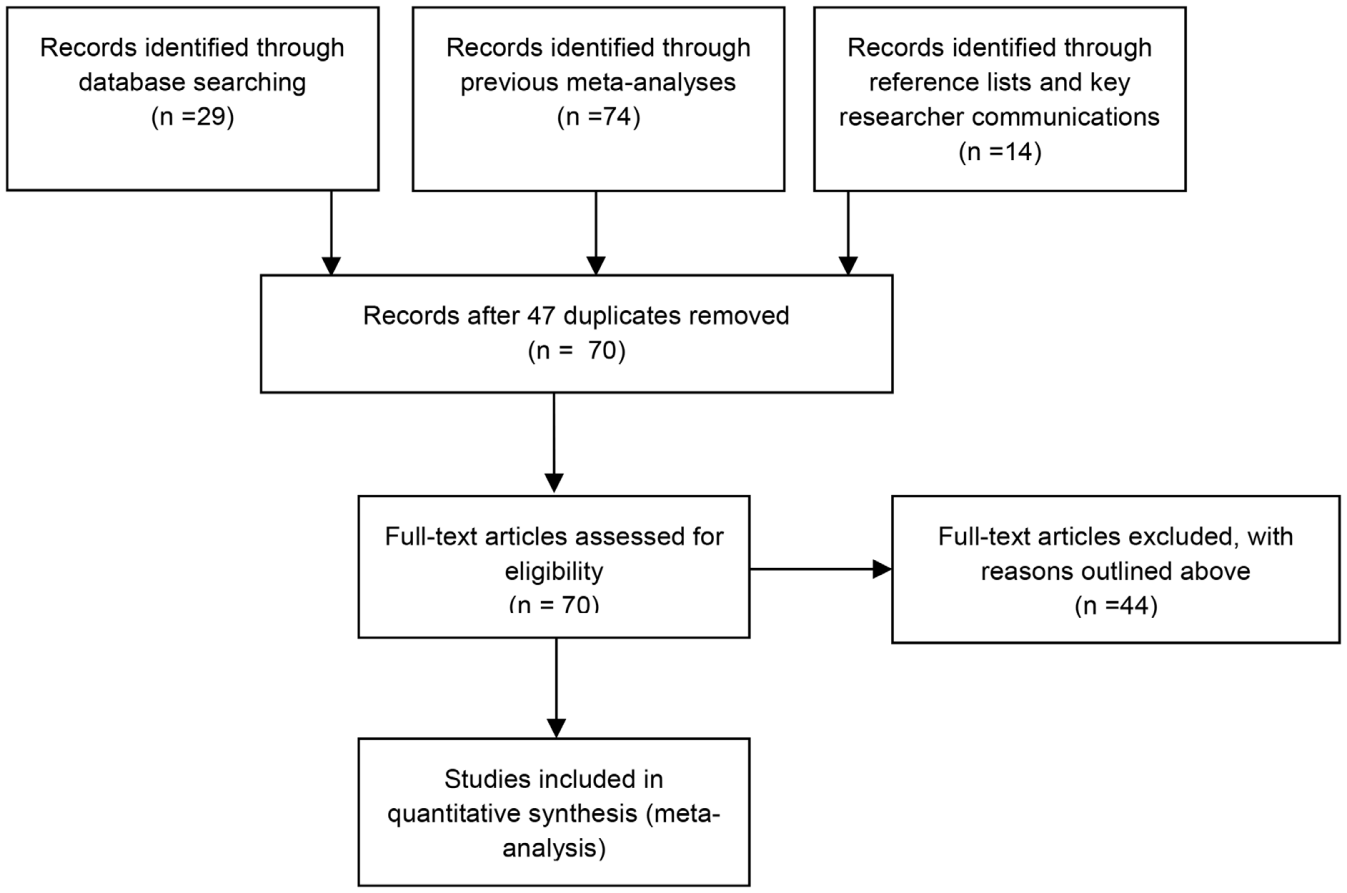
Flowchart of study inclusion.

Study details are presented in table 1 and inclusion flow chart figure 1.

**Table 1:** Properties of included studies.

Study FirstAuthor	Year	ControlType	BA	ClinicalInterview	OutcomeMeasure	Level ofTherapist	DeliveryMode	No ofSessions	BaselineDepression Level	Population	Country	Follow-upPeriod
Fuchs [65]	1977	WL	S	Other	SR	SP	G	6	Mod Severe	General	USA	6 weeks
Shaw [66]	1977	WL	S	Other	Cl/SR	SP	G	8	Mod Severe	University	USA	4 weeks
Taylor [67]	1977	WL	C	Other	SR	SP	I	6	Mod Severe	University	Canada	5 weeks
McLean [68]	1979	Placebo/medication	S	Other	SR	SP	I	10	Mod Severe	General	USA	12 weeks
Comas-Diaz [69]	1981	WL	S	Other	Cl/SR	SP	G	5	Mod Severe	General	USA	Posttreatment
Rehm [70]	1981	WL	S	Structured	SR	SP	G	7	Mod Severe	General	USA	7 weeks
M Lopez [71]	1982	TAU	S	Other	Cl/SR	SP	I	10	Mod Severe	General	Spain	Posttreatment/12weeks
Wilson [72]	1982	Placebo/WL/medication	S	Other	SR	SP	I	7	Mod Severe	General	Australia	1 week/24weeks
Wilson [73]	1983	WL	S	Other	Cl/SR	SP	I	8	Mod Severe	General	Australia	8 weeks
Skinner [74]	1984	WL	S	Other	SR	SP	I	5	Mod Severe	General	USA	5 weeks
Thompson [75]	1984	WL	S	Structured	Cl/SR	SP	I	6	Mod Severe	Older	USA	6 weeks
Thompson [76]	1987	WL	S	Structured	Cl/SR	SP	I	16	Mod Severe	Older	USA	6 weeks
Lovett [77]	1988	WL	S	Other	Cl	SP	G	10	Mild Mod	Older	USA	10 weeks
Van denHout [78]	1995	TAU	S	Structured	SR	SP	G	12	Mod Severe	General	Netherland	Posttreatment/12weeks
Rokke [79]	1999	WL	S	Structured	Cl	SP	I	10	Mild Mod	Older	USA	10 weeks
Gallagher [80]	2000	WL	S	Structured	Cl	SP	G	10	Mild Mod	General	USA	12 weeks
Study First Author	Year	Control Type	BA	ClinicalInterview	OutcomeMeasure	Level ofTherapist	DeliveryMode	No ofSessions	BaselineDepression Level	Population	Country	Follow-upPeriod
Cullen [81]	2006	WL	C	Structured	SR	SP	I	10	Mod Severe	General	USA	Posttreatment/12weeks
Dimijian [82]	2006	Placebo/medication	C	Structured	Cl/Self R	SP	I	16	Mild Mod/ModSevere	General	USA	8 weeks
Gawrysiak [83]	2009	WL	C	Other	SR	SP	I	1	Mod Severe	University	USA	2 weeks
Mitchell [84]	2009	TAU	S	Structured	Cl	NS	I	9	Mod Severe	General	USA	9 weeks/12months/24month
Ekers [85]	2011	TAU	C	Structured	SR	NS	I	12	Mod Severe	General	UK	12 weeks
Armento [86]	2012	Placebo	C	Structured	SR	SP	G	1	Mild Mod	University	USA	4 weeks
Carlbring [87]	2013	WL	C	Structured	SR	SP	SH	7	Mod Severe	General	Sweden	8 weeks
Kanter [88]	2013	TAU	C	Structured	Cl/SR	SP	I	12	Mod Severe	General	USA	Posttreatment/36weeks
Moradveisi [89]	2013	Medication	C	Structured	Cl/SR	SP	I	8	Mod Severe	General	Iran	4/13/49weeks
O’Mahen [90]	2013	TAU	C	Other	SR	NS	SH	8	Mod Severe	Women	UK	17 weeks/24weeks

### Description of Studies

Twenty five studies compared BA with control treatments with a total of 1088 subjects (BA condition N = 547; Control condition N = 541) matched the inclusion criteria and were included in the current meta-analysis. A summary of the characteristics of the included studies are presented in Table 1. Sixteen studies focused on the general population, four focused on university students, four focused on older adults, and one on women with post-natal depression. Nineteen studies were set in specialist mental health services, four in primary care or physical health care and two were web based. Sixteen studies involved participants contacting the research team, four studies used screening procedures, three studies used referral and two a mixed approach. Nine studies incorporated complex BA as an intervention and the remaining 16 incorporated simple BA. Twelve studies used a structured clinical interview whereas the remaining 13 used other unstructured forms. Ten studies used both clinician and self-rated measures of depression, 11 used only self-rated measures, and four used clinician rated measures. Treatment as usual was used for the control type in six of the studies, waiting list control was used in 15 of the studies, and a psychological placebo intervention was used in three of the studies. One study used both a waiting list and a placebo as control type. The level of therapist varied from specialist in 22 of the studies, and non-specialist in the remaining three. The delivery mode of the therapy was in an individual format in 15 of the studies, a group format in eight and self-help in the remaining two. Baseline depression scores were moderate to severe for 20 of the studies and mild to moderate for four. One study included both mild-moderate and moderate to severe scores. Number of sessions varied between one and 16. Seventeen studies were conducted in the United States, two in Australia, one in Canada, one in Sweden, one in the Netherlands, one in Spain, and two in the UK.

One additional study [89] and three studies also included in the BA vs. control comparison [68], [72], [82], [89] were included in the BA vs. Medication meta-analysis (BA condition n = 130; anti-depressant medication n = 153). Two studies used SSRI medication ad complex BA [82], [89] with the other two using tri-cyclic medication ad simple BA. Further details of these studies can be seen in table 1.

We generally classed studies as low quality with only seven reporting three or more of our quality standards (see table 2).

**Table 2:** Study quality assessment.

First Author	Year	Study Quality Elements (+/−)			
		Q1	Q2	Q3	Q4
**Fuchs [65]**	1977	−	−	−	−
**Shaw [66]**	1977	−	−	+	−
**Taylor [67]**	1977	−	−	−	−
**Mclean [68]**	1979	−	−	+	−
**Comas-Diaz [69]**	1981	−	−	−	−
**Rehm [70]**	1981	−	−	+	−
**Maldonado−Lopez [71]**	1982	−	−	−	−
**Wilson [72]**	1982	−	−	−	−
**Wilson [73]**	1983	−	−	−	−
**Skinner [74]**	1984	−	−	−	−
**Thompson [75]**	1984	−	−	−	−
**Thompson [76]**	1987	−	−	−	−
**Lovett [77]**	1988	−	−	−	−
**Van den Hout [78]**	1995	−	−	−	−
**Rokke [79]**	1999	−	−	−	+
**Gallagher-Thompson [80]**	2000	−	−	−	−
**Cullen [81]**	2006	−	−	−	+
**Dimijian [82]**	2006	+	−	+	+
**Gawrysiak [83]**	2009	−	−	−	−
**Mitchell [84]**	2009	+	+	+	+
**Ekers [85]**	2011	+	+	+	+
**Armento [86]**	2012	−	−	−	−
**Carlbring [87]**	2013	+	+	+	+
**Kanter [88]**	2013	+	+	+	+
**Moradveisi [89]**	2013	+	+	+	+
**O’Mahen [90]**	2013	+	+	−	+

Q1: Adequate generation of randomisation sequence; Q2: Allocation concealment; Q3: Blinding of assessment; Q4: dealing with missing data.

### Meta-Analysis BA vs. Control Interventions

**Figure 2:**
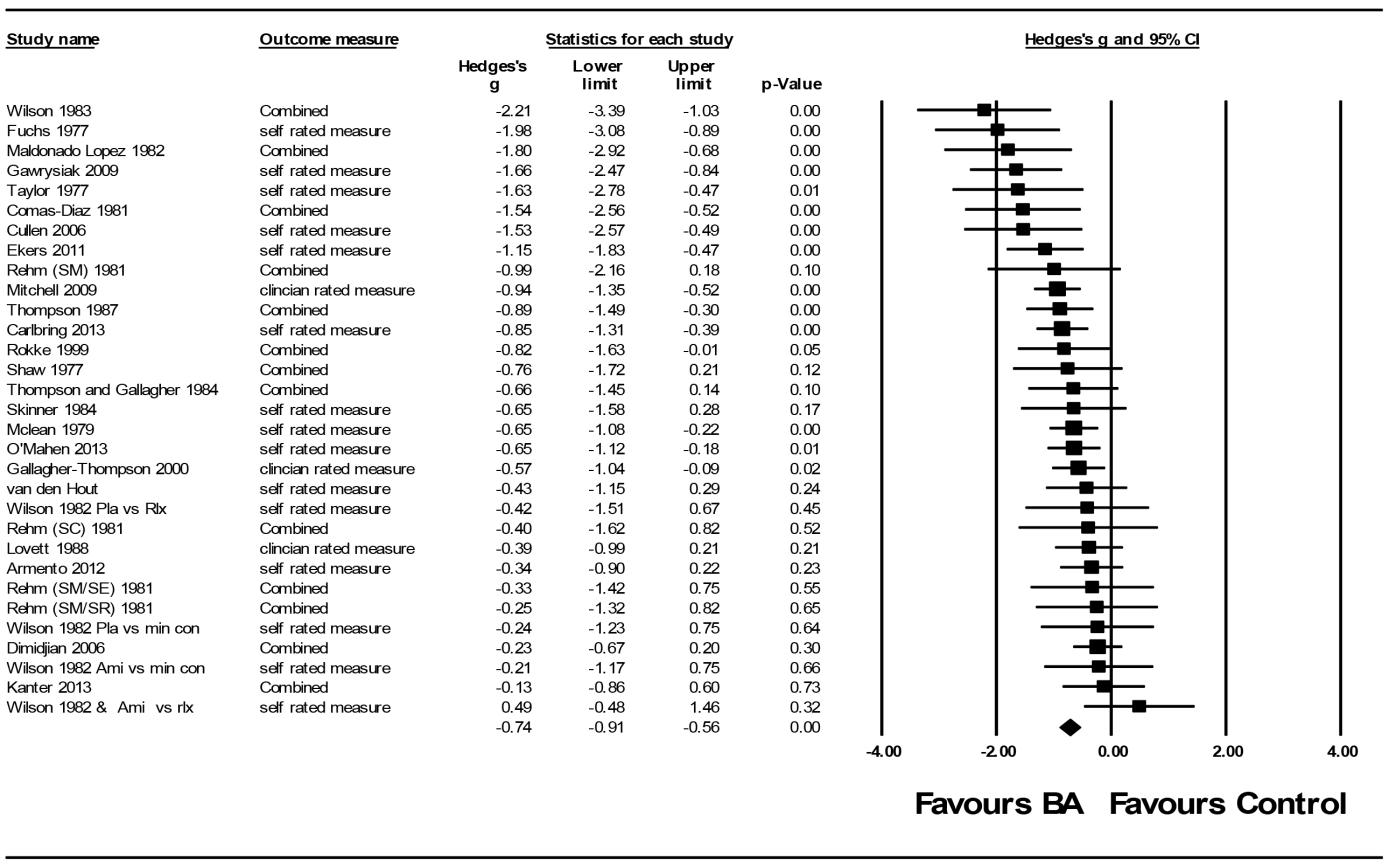
Behavioural Activation vs. control post treatment (ordered by effect size high to low).

BA for depression was compared to controls in 25 studies including 31 comparisons and 1088 participants. The SMD (*g*) at post treatment was −0.74 (95% CI −0.91 to −0.56 *p*<0.001 NNT 2.5), representing a large effect size (fig. 2). Sensitivity analysis replacing mid-range imputed standard deviations with lowest and highest observed values had minimal influence on results (*g* = −0.89, 95% CI −1.14 to −0.64 and *g* = −0.67 95% CI −0.83 to −0.50 respectively). There was moderate between-study heterogeneity of treatment effects beyond what would be expected due to sampling error (Q 51.64 p 0.008 I^2^ 41.91%). Subgroup analysis was used to explore this dispersion further. We found a significant association with effect size and subgroup in two areas, control type and baseline depression severity. All other subgroup comparisons identified similar SMD across groups (see table 3). Study quality was sub optimal in all but six studies, subgroup analysis indicated no significant relationship between study quality and effect size. The SMD (*g*) of comparisons in low quality studies at post treatment was −0.77 and in high quality studies −0.67 with similar levels of statistical heterogeneity (see table 3). The median number of clinical sessions with a therapist was eight (range one to 16). Meta-regression using session number as a mediator resulted in a slope of 0.03 (95% CI −0.01 to 0.06, Q _total_ 51.92 p = 0.01, Q _session number_ 2.08 p = 0.15), indicating no significant influence on effect size. Meta-regression using BA components as a mediator resulted in a non-significant slope of 0.04 (95% CI −0.11 to 0.20, Q _total_ 51.64 p = 0.01, Q _session number_ 0.32 p = 0.57), indicating minimal influence on effect size.

**Table 3:** Behavioural Activation vs. control effect size and subgroup analysis^a^.

		Number ofcomparisons	SMD (g)	95% CI	I^2^	I^2^ 95% CI(P^b^)	P (betweensubgroups)	NNT
BA vs. Control								
All Studies post treatment		31	−0.74	−0.91 to −0.56****	41	11 to 62**		2.50
One effect size per study highest		25	−0.80	−1.00 to −0.61****	49	20 to 68***		2.34
One effect size per study lowest		25	−0.80	−0.99 to −0.61****	44	12 to 66**		2.34
Self-rated measures only		28	−0.75	−0.96 to −0.54****	48	20 to 67***		2.48
Clinician rated measures only		16	−0.73	−0.96 to −0.49****	35	0 to 65*		2.54
All studies 6–9 month Follow up		8	−0.35	−0.59 to −0.11***	0	0 to 68		5.1
*Subgroup Analysis*								
Study population	General Adult	23	−0.72	−0.95 to −0.49****	48	15 to 69**	0.83	2.56
	Older Adult	4	−0.68	−1.02 to −0.34****	0	0 to 85		2.70
	Post natal	1	−0.65	−1.12 to −0.18****	0	n/a		2.82
	Student	4	−1.03	−1.75 to −0.31****	65	0 to 88***		1.87
Recruitment Setting	Specialist Mental Health	25	−0.73	−0.97 to −0.50****	49	19–68***	0.93	2.54
	Physical/Primary Care	4	−0.80	−1.17 to −0.50****	11	0–86		2.34
	Online	2	−0.75	−1.08 to −0.42****	0	n/a		2.48
Recruitment Process	Screening	4	−0.82	−1.19 to −0.45****	39	0–79	0.91	2.28
	Volunteer	22	−0.69	−0.91 to −0.47****	46	11–67***		2.67
	Referral	3	−0.90	−1.71 to −0.08**	68	0–91**		2.10
	Mixed	2	−0.80	−1.41 to −0.176**	0	n/a		2.34
Baseline Depression Severity	Mild- mid moderate	5	−0.41	−0.67 to −0.14***	0	0 to 79	0.02	4.39
	Mid moderate-severe	26	−0.82	−1.00 to −0.61****	40	8 to 64**		2.28
Diagnostic interview	Yes	15	−0.88	−1.22 to −0.54****	58	27 to 77***	0.23	2.15
	No	16	−0.65	−0.82 to −0.48****	9	0 to 47		2.82
Mode of delivery	Group	11	−0.62	−0.89 to −0.35****	17	0 to 58	0.64	2.96
	Individual	18	−0.80	−1.06 to −0.53****	55	24 to 74***		2.34
	Facilitated Self help	2	−0.75	−1.08 to −0.42****	0	n/a		2.48
Therapist Level	Non-Specialist	3	−0.87	−1.15 to −0.59****	0	0 to 90	0.38	2.16
	Specialist	28	−0.71	−0.91 to −0.51****	44	13 to 64**		2.60
Intervention Complexity	Simple	22	−0.71	−0.92 to −0.51****	32	0 to 60*	0.70	2.60
	Complex	9	−0.79	−1.14 to −0.45****	61	20 to 81***		2.36
Control Type	Waiting list	20	−0.87	−1.10 to −0.64****	32	0 to 61*	0.20	2.16
	TAU	6	−0.78	−1.13 to −0.43****	45	0 to 78		2.39
	Placebo Intervention	5	−0.34	−0.64 to −0.05**	19	0 to 83		5.26
Quality of Study	High (3≥)	6	−0.67	−0.96 to −0.37****	50	0 to 80	0.58	2.75
	Low (3<)	25	−0.77	−0.99 to −0.5****	42	7 to 64		2.42
BA vs. Anti-Depressant medication								
All Studies		4	−0.42	−0.83 to −0.00**	64	0 to 88**		4.27
Drug Type	SSRI	2	−0.38	−1.24 to 0.47	87	n/a	0.82	4.72
	Tri-cyclic	2	−0.49	−0.87 to −0.11**	0	n/a		3.68

Abbreviations: Standardised Mean Difference SMD (g): Confidence Interval CI; Numbers Needed to Treat NNT, Treatment as Usual TAU,

a Hedges g, ^b^ p values in this column indicate if Q statistic is significant (I^2^ does not provide test of significance).

*p<0.10.

** p<0.05.

*** p<0.005.

****p<0.0005.

Inspection of the funnel plot indicated no evidence of publication bias. Trim and fill procedures supported this observation, suggesting no change in effect sizes when imputation for potential missing data was undertaken. Egger’s test indicated a symmetrical distribution (intercept −0.92 95% CI −2.26 to 0.43 p = 0.17). In 13 (50%) studies with the largest sample sizes an SMD −0.62 (−0.78 to −0.47) was observed indicating only a limited influence of small studies on the overall estimated effect.

Five studies including eight comparisons and 273 participants provided follow up data between 6–9 months. The SMD (*g*) at follow up was −0.35 (95% CI −0.59 to −0.11 *p*<0.001 NNT 5.1), representing a medium effect size. There was no evidence of between-study heterogeneity of treatment effects (Q 5.12, p 0.66, I^2^ 0%).

### Meta-analysis BA vs. Antidepressant Medication

**Figure 3:**
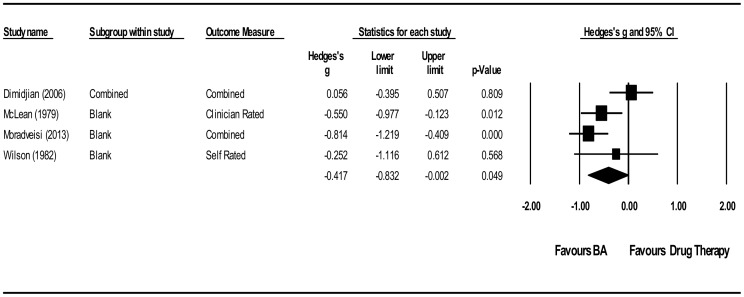
Behavioural Activation vs. Antidepressant medication.

BA for depression was compared to antidepressant medication in four studies including 283 participants. The SMD (g) at post treatment was −0.42 (95% CI −0.83 to −0.00 *p* 0.05 NNT 4.27), representing a moderate effect size in favour of BA (see fig. 3). There was moderate between-study heterogeneity of treatment effects beyond what would be expected due to sampling error (Q 8.34 p 0.04, I^2^ 64.02%). Two studies used SSRI [82], [89] with two studies tricyclic antidepressant medication [68], [72] with no apparent association between drug type and effect size (see table 2). There were insufficient studies to allow further exploration of subgroups or potential publication bias. We conducted sensitivity analysis on study quality by removing the two low quality studies from the analysis [68], [72] resulting in a non-significant effect size in favour of BA of −0.38 (95% CI −1.23 to 0.47 *p* 0.38).

## Discussion

In this updated review we found that behavioural activation for depression is clinically effective. With the increased interest in BA over previous years such an update was needed as our previous reviews were conducted over 5 years ago [5], [6]. This current review includes 26 studies which is a clear increase over the 16/17 included in those previous reviews. In addition this current update addresses some of the gaps identified in those reviews (BA vs. medication). We found BA to be superior to controls across 31 comparisons in 25 studies and small but significant short term superiority to antidepressant medication.

We found a large effect size across studies (g−0.74), similar to those found in our previous reviews (d = −0.70 and −0.87 respectively) whilst including considerably more comparisons and participants. The confidence intervals around these results have decreased slightly from previous reviews. However it is of note that generally studies were small, of low quality and results were short term. This is not surprising as psychotherapy studies often include small sample sizes and participants in control arms were also often offered active treatment after a delay period. We have, however, been able to include sufficient studies in our meta-analysis providing a good overview of findings and the ability to explore the moderate heterogeneity found by subgroup analysis.

We explored the association between the types of participant recruited and the effect size of the intervention in three subgroup comparisons. We could find no difference in effect between recruitment groups (general adult, older adult, student, post natal) nor if a diagnostic interview had been used in studies, although statistical power to detect differences between subgroups was low. We did however find a larger effect size in studies that had higher baseline depression severity. In addition the setting within which recruitment was conducted and the processes used to identify participants did not moderate the effect size of BA.

Intervention factors appeared to have no association with effect size. Most studies used individual face to face or group therapy with two studies using self-help based BA with a comparable effect size across delivery modes. One of the potential benefits of BA that has been discussed for some time has been the potential for dissemination due to the relative simplicity of the treatment [29]. In our previous meta-analysis we found no evidence to support this claim, however in this review three studies did include non-specialist therapists. The effect sizes in these studies were large and consistent, and no different from those seen in studies using specialists. Despite being few in number, studies using non-specialists were well conducted and no heterogeneity was observed between them, providing the first evidence supporting the dissemination of BA outside expert delivery. In addition we considered the complexity of BA, observations that are timely as recently some reviewers have sought to reclassify complex BA approaches as a third wave CBT distinct from core BA elements [9]. We found no association between effect size and the level of complexity of the BA used in studies where functional analysis and other ‘complex’ elements were added; as such the re-branding of a sub set of BA studies would appear premature. In addition to the complexity we explored the number of sessions via meta-regression. The median number of sessions in included studies was eight, there was no evidence that the number of sessions was associated with effect size.

BA was compared to a waiting list control in 20 comparisons, usual care in six and a placebo intervention in five. A significant effect was found indicating that the effect size in those studies using a placebo intervention (attention control/relaxation/drug placebo) as control were smaller than those using waiting list or usual care.

In summary we found no evidence that population, approach to clinical diagnosis, number of sessions or therapist qualification/complexity of BA had any association with outcome. We did however observe a relationship between baseline severity and the type of control group with outcome. The degree to which this explains the overall heterogeneity observed in our main post treatment results is unclear but the findings provide some analysis of that finding.

Previous reviews have not included a meta-analysis of BA vs. medication due to the limitation of available evidence, NICE [1] reported one study indicating no difference between groups. In this review we were able to include four studies and found a small but significant difference at post treatment in favour of BA. It is of note, however, when low quality studies were removed from the analysis these differences disappeared suggesting caution when interpreting results. There appeared to be no difference between types of anti-depressant, but again both studies that use tri cyclic medication were of low quality limiting reliability of findings.

A number of limitations exist to this review. Whilst we were able to include a reasonable number of studies it is of note that many were small and of poor quality. The median sample size in the BA arms were 11 and 16 for controls and medication groups, with ranges of 4 to 56 and 9 to 50 respectively. This links directly to the quality of the studies, there were a significant amount of older studies which generally were not subject to the same level of quality standards as those conducted in recent years. Rather than exclude such studies we chose to include them and deal with quality issues via subgroup and sensitivity analysis. Whilst study quality was not associated with effect size when BA was compared to controls it is of note that only seven studies of the 26 included met three or more commonly accepted standards for RCTs. Study quality appears to be improving over time with those seven studies being generally the most recently conducted however the publication of further high quality studies is needed to improve confidence in these findings. In contrast when poor quality studies were excluded in the BA comparison to medication analysis, the significance of the effect in favour of BA disappeared. This suggests that results found in this comparison must be viewed with caution due to the limited numbers of studies and participants included in the review. We focus mainly on depression outcomes post treatment as only five studies include follow up data beyond 6 months. Some other studies do report longer term follow up for BA that appears promising [38] however comparisons are with other active therapeutic interventions, not control participants, and as such did not meet our inclusion criteria. Our analysis of follow up data vs. control interventions indicates a medium effect size between six and nine months however further research is required examining the longer term benefits of BA. Seventeen of the 26 included studies were conducted in the United States (US) and whilst we could observe no difference between the effect sizes between those inside and outside the US this should be considered in the interpretation of results. The key argument linked to the dissemination of BA is the durability within wider dissemination and whilst we were able to conduct the first exploration of this in meta-analysis from a clinical perspective the linked question of cost utility requires more research.

Despite limitations, our updated meta-analysis provides evidence that supports BA as an effective treatment for depression with outcomes at least as effective as anti-depressant medication. We have found early indications supporting the implementation of the intervention beyond the traditional psychotherapy workforce. Further, individually fully powered and high quality trials are needed to test BA in terms of low cost implementation and the cost effectiveness this may offer. We are aware of at least one large scale randomised controlled trial currently underway to answer these questions [91].

## Supporting Information

Checklist S1 PRISMA Checklist.
(DOC)
